# Poster Session I - A109 REPRODUCTIVE CARE DURING PRECONCEPTION AND PREGNANCY IN INFLAMMATORY BOWEL DISEASE (IBD): IMMIGRANT VS. NON-IMMIGRANT PERSPECTIVES

**DOI:** 10.1093/jcag/gwaf042.109

**Published:** 2026-02-13

**Authors:** C Gafrey, V W Huang, K O’ Connor, P Tandon

**Affiliations:** Medicine, University of Toronto, Toronto, ON, Canada; University of Toronto, Toronto, ON, Canada; Medicine, University of Toronto, Toronto, ON, Canada; Medicine, University of Toronto, Toronto, ON, Canada

## Abstract

**Background:**

Women with IBD face an increased risk of adverse pregnancy outcomes compared to those without IBD. They are known to have increased healthcare utilization, including more frequent hospitalizations and are more likely to have inadequate prenatal care, which is associated with adverse pregnancy outcomes.

**Aims:**

This study aims to identify gaps in perinatal care between immigrant and non-immigrant women with IBD to inform the development of culturally appropriate care.

**Methods:**

We recruited women with and without an immigration history aged 18 or over with IBD to anonymously complete an online survey that was distributed throughout ambulatory clinics and on social media platforms. Descriptive statistics were reported as frequencies, and cross-group comparison was completed using the chi-square test.

**Results:**

We included 53 immigrant and 169 non-immigrant women. Baseline demographics were similar between groups. Most immigrant women had Ulcerative Colitis (56%) vs non-immigrants who primarily had Crohn’s Disease (53%) p = <0.001. Fewer immigrants had prior pregnancies (20(37.7%) vs 72(42.6%) p = <0.001). Of those who had been pregnant, there was no difference in the number of miscarriages or abortions between the groups (p = 0.97). Many were in the preconception stage as 12 (23%) vs 30 (18%) were currently pregnant p < 0.001.

Immigrant-specific questions indicated that 15(28.3%) went back to their home country to get care, despite 34 (64%) being diagnosed after relocation. 7(13%) considered going back to their home country due to challenges faced due to their IBD vs 4 (8%) for reproductive care. Free-text responses on reproductive care highlighted themes of limited information, longer wait times, reduced specialist access, fewer tests, and decreased support after relocation. 6 (11%) were concerned about how their home foods could cause flares, compared to 30 (57%) who were concerned about westernized foods causing flares (p < 0.001).

Communication-related questions revealed that more immigrants did not speak the same language as their GI doctor (19 (35%) vs 1 (0.6%) p < 0.001). Despite this, 15 (28%) did not have access to formal translation materials. Immigrant women were less likely to discuss family planning with their GI doctor compared to non-immigrants (30(57%) vs 122 (72%) p < 0.001).

Immigrant women were less likely to feel confident in their reproductive care (6 (11%) vs 11 (7%) p < 0.001). Regarding information seeking, most women sought information through their GI (34(64%) vs 117 (69%) p < 0.001). There was no difference in the number of women seeking care on social media (13% vs 10%).

**Conclusions:**

Immigrant women with IBD experienced barriers to reproductive care, including communication challenges, reduced access to specialists, and limited culturally tailored support.

**Funding Agencies:**

None

